# Discovery of MAGL Inhibition by Lophine Derivatives: An Unexpected Finding from Chemiluminescent Assay Development

**DOI:** 10.3390/molecules30071605

**Published:** 2025-04-03

**Authors:** Roberta Ottria, Silvana Casati, Ornella Xynomilakis, Aleksandar Veselinović, Pierangela Ciuffreda

**Affiliations:** 1 Dipartimento di Scienze Biomediche e Cliniche, Università degli Studi di Milano, 20157 Milano, Italy; silvana.casati@unimi.it (S.C.); ornella.xynomilakis@unimi.it (O.X.); pierangela.ciuffreda@unimi.it (P.C.); 2 Department of Chemistry, Faculty of Medicine, University of Niš, Blvd. Dr. Zorana Đinđića 81, 18000 Niš, Serbia; aveselinovic@medfak.ni.ac.rs

**Keywords:** lophine derivatives, monoacylglycerol lipase, MAGL, chemiluminescence assay, enhancers, inhibitors, docking studies

## Abstract

The inhibitory effects of two novel lophine derivatives were unexpectedly discovered during the development of a chemiluminescent monoacylglycerol lipase (MAGL) assay. The proposed lophine derivatives were found to exhibit concentration-dependent inhibitory effects on MAGL with the octanoic and palmitic acid esters of 2-(4-hydroxyphenyl)-4,5-diphenylimidazole showing the strongest activity. Reversibility assays using a fluorometric method confirmed that these compounds interact with MAGL in a stable, irreversible manner. To further investigate their mode of interaction, docking studies were performed, supporting the hypothesis that compounds **3** and **4** may act as competitive and irreversible inhibitors. Lophine derivatives were initially designed and synthesized as potential chemiluminescence pro-enhancers. However, assay optimization revealed no signal production upon MAGL hydrolysis, precluding their use as chemiluminescent probes. These findings suggest that lophine is a promising candidate for the development of MAGL inhibitors, although further optimization is needed to enhance binding affinity and selectivity.

## 1. Introduction

The endocannabinoid system [1] is a widespread signaling network that plays a key role in various physiological and pathological processes [2,3,4,5]. Its dysregulation has been linked to neurological, metabolic, and cardiovascular disorders, as well as tumor development [6,7,8,9]. This makes ECS modulation a promising target for diagnostic and therapeutic approaches, either through genetic or pharmacological interventions [10,11]. Drug design programs have targeted various endocannabinoid system components to treat diseases affecting both the central and peripheral systems [12,13]. A promising approach is regulating endocannabinoid levels by inhibiting hydrolytic enzymes, allowing precise control of their activity while minimizing psychotropic side effects [14,15,16,17]. In this field, the study of 2-arachidonoylglycerol levels and the possibility of controlling them through the inhibition of its principal hydrolytic enzyme, monoacylglycerol lipase (MAGL), has attracted significant interest [16,18,19,20,21,22]. Over the years, several classes of inhibitors have been designed to modulate MAGL activity, including sulfonyl fluorides (I), fluorophosphates (II), trifluoromethyl ketones (III), carbamates (IV), and ureas (V) [23,24]. Of note, the irreversible inhibition or chronic high dosing of MAGL inhibitors is associated with negative effects such as physical dependence, pharmacological tolerance or desensitization, and the downregulation of cannabinoid receptor 2. Consequently, it has been hypothesized that the use of reversible selective and potent inhibitors could avoid these adverse effects. However, the irreversible inhibition of MAGL could find application at the peripheral and local levels [12,25]. While current methods for assessing MAGL activity, such as LC/MS and HPLC, offer precise measurements, they require expensive instrumentation, making them unsuitable for high-throughput screening (HTS) [26]. Luminometric techniques, like fluorometric methods, are practical for assessing MAGL activity and are well suited for HTS with common laboratory equipment. These methods use acid esters of known fluorophores as substrates, which MAGL hydrolyzes to produce free fatty acids and fluorescent products [27]. Substrates like 7-hydroxycoumarinyl-arachidonate (7-HCA) [28] and 7-hydroxyresorufinyl octanoate (7-HRO) [29] are efficient for HTS, but fluorescent methods often suffer from high background signals, limiting their use in cell cultures or in vivo studies. To overcome fluorescence limits, this research project aims to develop a novel chemiluminescence (CL)-based method to assess MAGL activity, combining high sensitivity with rapid performance [30]. CL methods offer distinct advantages over fluorescence or LC/MS techniques, including superior sensitivity, faster processing times, and minimal background interference [31]. These attributes make CL particularly well suited for both in vitro and in vivo applications as a robust alternative to conventional colorimetric and fluorometric methods for HTS. Moreover, cost-effective instrumentation and minimal background interference are particularly advantageous for detecting low biomolecule concentrations [32] in enzyme activity studies, immunoassays, and diagnostic applications. Horseradish peroxidase (HRP) is one of the enzymes most widely used in CL for analytical biochemistry applications, owing to its critical role in enabling highly sensitive detection methods. HRP is commonly employed for detecting hydrogen peroxide (H_2_O_2_) and other compounds through coupled enzymatic reactions. A well-established CL method involving HRP is the oxidation of luminol in the presence of H_2_O_2_, which generates a chemiluminescent signal [33]. Although the precise mechanistic details and the identity of individual light-emitting species are not fully understood, the peroxidase-catalyzed chemiluminescent oxidation of luminol has been extensively studied. The process is generally recognized as a multi-step reaction: luminol is oxidized via a complex formed between the oxidant and HRP, producing a luminol radical. The subsequent involvement of metals facilitates the combination of molecular oxygen with the enol form of the luminol anion, leading to the formation of a cyclic peroxide intermediate. This intermediate decomposes into 3-amino-1,2-benzenedicarboxylic acid in an excited state and a molecule of nitrogen is released. Finally, the emission of a photon of blue light follows the 3-aminophthalate drop down [34].

Traditional CL assays for peroxidase activity are limited by weak signals, poor signal-to-background ratios, low-intensity light emission under interference-minimized conditions, and rapid luminescence decay. To improve CL methods, signal enhancers have been incorporated into CL HRP–luminol assays. These mediators improve signal strength, extending luminescence duration and ensuring stable light emission without altering the chemical properties of the final products [35]. Effective enhancers include substituted phenols, such as 6-hydroxybenzothiazole derivatives [36,37], and aryl-boronic acids like 4-iodophenylboronic acid [38], 4-iodophenol [39], and 4-(4-hydroxyphenyl) thiazole [40]. Studies suggest a “ping-pong” mechanism involving the simultaneous oxidation of luminol and the enhancer, driven by enzyme-catalyzed reactions. Lophine (2,4,5-triphenyl-1H-imidazole) is a fluorescent and chemiluminescent compound first described by Radziszewski in 1877 [41]. In addition to its optical and CL properties, it emits yellow light upon reaction with oxygen under basic conditions. This characteristic, coupled with its relatively straightforward synthesis from readily available and inexpensive reagents, has contributed to its enduring appeal in various research fields. Since its initial discovery, a wide array of lophine derivatives have been synthesized and investigated [33], primarily for analytical applications in detecting trace amounts of compounds in biological matrices [34,42]. While methods for evaluating enzyme activity modulation are valuable for researchers, the range of techniques specifically designed to assess MAGL activity remains relatively limited in the current literature. Lophine’s established utility in analytical chemistry, combined with its inherent chemiluminescent potential, prompted us to design and synthesize novel lophine ester derivatives. A lophine ester derivative was studied to develop a chemiluminescent method to assess the activity of lipases such as *Candida cylindracea* lipase and *Porcine pancreas* lipase [43]. We synthesized three esters with chain lengths of 4, 8, and 16 carbon atoms based on our previous knowledge on MAGL hydrolysis preference [29]. These derivatives are envisioned as promising chemiluminescent probes specifically tailored for the development and validation of a highly sensitive method for evaluating MAGL activity.

We hypothesized that the unique properties of lophine, when incorporated into appropriate ester structures, could provide a sensitive and reliable tool to quantify MAGL activity, opening new avenues for studying this important enzyme.

However, our studies unexpectedly led us in a different direction, resulting in the discovery of MAGL inhibitors. For this reason, we also present here the application of a fluorescence-based method for the comprehensive characterization of three compounds, two of which are novel, as MAGL inhibitors. Finally, docking studies are reported to elucidate the interaction mechanism between lophine derivatives and MAGL.

## 2. Results and Discussion

Motivated by the need for more sensitive MAGL assays [29,44,45,46], we explored CL due to its simplicity, low cost, and superior sensitivity [47,48]. CL offers advantages for cell-based assays by eliminating external excitation, thus avoiding cellular damage, auto-fluorescence background, and issues common in fluorescence-based methods [49]. Furthermore, signal amplification via potentiators improves CL detection. Therefore, this study investigates a novel CL-based MAGL assay, employing the luminol–H_2_O_2_–HRP reaction with a 2-(4-hydroxyphenyl)-4,5-diphenylimidazole (HDI) ester pro-enhancer substrate, which releases the active enhancer HDI upon enzymatic hydrolysis.

### 2.1. Synthesis of HDI *(**1**)* and Lophine Derivatives ***2***–***4***

**Scheme 1:**
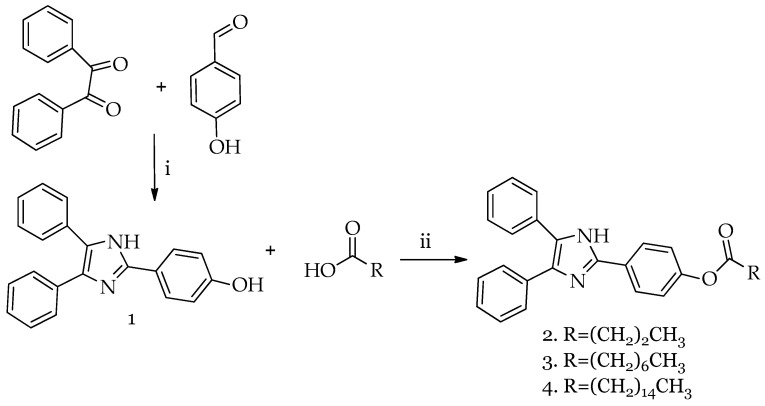
Synthesis of compounds **1**–**4**. Reagents and conditions for HDI (compound **1**) (i): acetic acid, ammonium acetate, reflux, 48 h; compounds **2**–**4**: (ii) DCC, DMAP, dry CH_2_Cl_2_, argon, reflux, 3 h.

Three lophine derivatives with varying chain lengths (C4, C8, C16), selected for their high chemical stability, low cost, and MAGL substrate similarity, were designed and synthesized. The synthesis began with the preparation of the enhancer HDI, following a modified reported procedure [50]. Briefly, diphenylethanedione reacted with 4-hydroxybenzaldehyde in the presence of acetic acid and ammonium acetate. Pure product was obtained via recrystallization in a 96% yield. Esters **2**, **3**, and **4** were subsequently synthesized following the literature procedure [51]. The coupling of compound HDI with the different acids was achieved after acid activation using dicyclohexylcarbodiimide in the presence of 4-dimethylaminopyridine (Scheme 1). Purification via column chromatography provided pure products as white solids with yields of 76%, 63%, and 69% for compounds **2**–**4**, respectively.

### 2.2. Setup of HDI–Luminol–H_2_O_2_–HRP System Conditions and Chemiluminescence Emission of Lophine Derivatives

**Figure 1:**
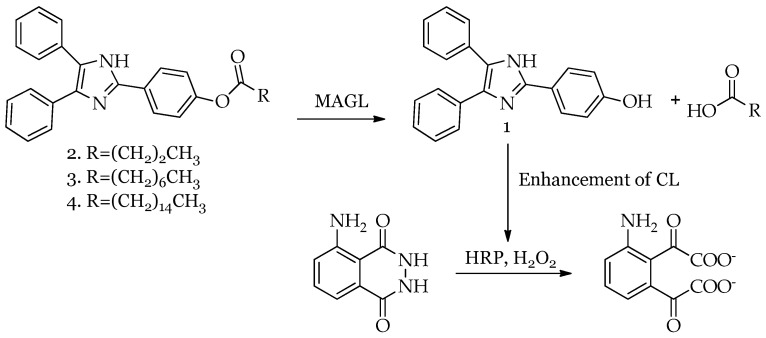
Enhancement CL mechanism of luminol–HRP–H_2_O_2_ system in the presence of lophine derivatives.

A luminometric assay was developed to assess MAGL activity based on a reported method [52]. In this assay, HDI esters (**2**–**4**) act as a pro-enhancer, which is hydrolyzed by MAGL to release active HDI and the corresponding carboxylic acid. The released HDI enhances luminescence in the luminol–H_2_O_2_–HRP system (Figure 1).

**Figure 2:**
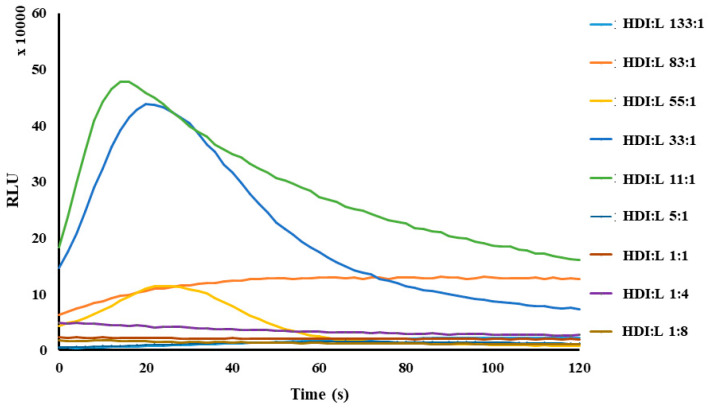
Time course of chemiluminescence emission obtained by varying the concentration ratios of **1** and luminol.

For the development of the chemiluminescent method, studies were conducted on HDI, as it represents the actual enhancer. The optimization of the assay focused on the reaction involving HDI, luminol, HRP, in Tris-HCl buffer (pH 7,4), and H_2_O_2_ sequentially. Since the luminometric signal is dependent on the amount of HDI released by MAGL, careful optimization was performed to define the detectable signal range. Optimal conditions were determined as 0.9 µM luminol, 1.7 × 10^−4^ HRP units, and 2.5 µL H_2_O_2_ per well. Various enhancer-to-luminol ratios (133:1 to 1:8) were tested (Figure 2). Optimal luminescence was observed at a 33:1 enhancer-to-luminol ratio (Figure 2 light blue)**,** confirming the previous literature findings that enhancers increase light emission up to an ideal concentration, beyond which additional amounts lead to a decrease in light intensity. Specifically, no detectable luminescence peak was observed for all luminol-to-enhancer ratios of 1:1 or greater (i.e., where luminol was in excess).

To optimize the MAGL-mediated pro-enhancer hydrolysis, the first reaction (MAGL with pro-enhancer) was optimized to maximize the method response. The interaction between MAGL and the pro-enhancers (compounds **2**, **3**, and **4**) was evaluated by varying the concentrations of the pro-enhancers (1 μM to 62,5 μM), MAGL (2.5 ng to 100 ng), and incubation time (0 to 60 min), while maintaining the optimized constant conditions of the second reaction (luminol–H_2_O_2_–HRP). Synthesized compounds were incubated with human recombinant MAGL (*h*MAGL) in reaction buffer (50 mM Tris-HCl, pH 7.4, 1 mM EDTA) at various times. Following incubation, luminol and H_2_O_2_ were added, and the mixture was transferred to wells containing HRP. To ensure compatibility with simple microplate readers (without the need for auto-injectors), HRP was added directly to the wells. However, despite these optimization efforts, no enhancement was obtained in CL intensity in the presence of compounds **2**–**4** compared to controls without hMAGL (Figure S1).

Two hypotheses were proposed to explain the lack of observed chemiluminescence enhancement. The first hypothesis centers on the possibility that the synthesized compounds (**2**, **3**, and **4**) are not acting as substrates for MAGL. This could be due to structural incompatibilities between the compounds and the enzyme’s active site: (i) the probe is not able to reach the active site; (ii) the probe positioning in the active site does not lead to the hydrolysis of the ester bound. The second hypothesis posits that the compounds could be irreversibly bound to the enzyme, effectively blocking its catalytic site and thus inactivating MAGL. All these mechanisms would prevent the enzyme from performing its normal function, which is necessary for the subsequent steps leading to chemiluminescence. To differentiate between these possibilities, further investigations were undertaken. A well-established fluorescence-based HTS assay for the evaluation of MAGL activity in the presence of enzyme modulators was employed to directly assess the interaction of the synthesized derivatives with hMAGL. Complementing these experimental studies, molecular docking studies were also performed. These computational studies aimed to model the interaction between the compounds and hMAGL at a molecular level. The docking simulations provided insights into the binding pose and affinity of the compounds for the enzyme, helping to determine whether they bind within the active site (suggesting inhibition) or elsewhere (potentially indicating a lack of substrate activity). The combined results from the fluorescence assays and docking studies were expected to shed light on the true mechanism of interaction between the synthesized compounds and hMAGL, ultimately explaining the absence of chemiluminescence enhancement.

### 2.3. Fluorometric Assay of Lophine Derivatives ***1***–***4***

To investigate whether compounds **2**–**4** could act as MAGL inhibitors, we studied the interaction of compounds **1**–**4** with MAGL, using a fluorometric assay based on a validated procedure [29,45]. This approach was chosen due to its previous application in HTS studies in potential MAGL inhibitor evaluations. The assay is based on the hydrolysis rate of 7-hydroxyresorufinyl octanoate (7-HRO), a fluorogenic probe, by MAGL, leading to the release of the fluorophore resorufine (excitation wavelength of 572 nm and an emission wavelength of 595 nm) [53]. The experimental setup involved incubating MAGL with varying concentrations of compounds **1**–**4** (50 nM–100 µM), followed by the addition of 7-HRO to initiate a kinetic analysis. Rigorous controls were implemented to account for the potential auto-hydrolysis of the probe or interference from the compounds themselves. Control samples to exclude auto-hydrolysis or compound interference included samples containing 7-HRO alone, MAGL alone, or compounds **1**–**4** alone in the reaction buffer. Fluorescence was monitored over time, and the resulting data were analyzed to determine the impact of each compound on MAGL activity.

**Figure 3:**
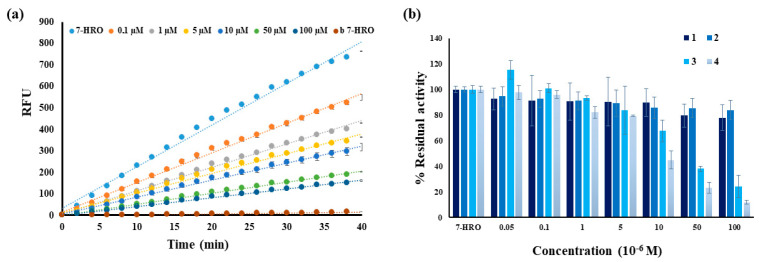
Fluorometric experiments results: (**a**) linear regression curves of kinetic experiment performed with compound **3** at different concentrations—b 7-HRO serves as the control for probe auto-hydrolysis; (**b**) residual activity percentage for compounds **1**–**4** at different concentrations (50 nM–100 µM).

Each experiment was performed in triplicate and repeated at least twice. The results were plotted as relative fluorescence units (RFU) vs. time and analyzed using linear regression. Residual enzymatic activity was calculated from the slope of the obtained curve, comparing MAGL activity in the presence or absence of potential inhibitors. Enzyme activity in presence of 7-HRO was considered to be 100%. The variation in fluorescence at the set wavelength of the compound **3** kinetic experiment is reported as an example of the performed experiments in Figure 3a. Residual activity in the presence of different concentrations of compounds **1**–**4** was calculated to assess the extent of inhibition or activation. The obtained mean residual activity percentages at different concentrations, together with standard deviations for compounds **1**–**4,** are plotted as histograms and are reported in Figure 3b.

Our results demonstrate that all tested compounds interact with MAGL, leading to a concentration-dependent reduction in enzyme activity. This observation suggests that these compounds act as inhibitors of MAGL, albeit with varying degrees of potency. Notably, compounds **3** and **4** exhibited the most pronounced inhibitory effects, suggesting a stronger interaction with the enzyme’s active site or a more effective disruption of its catalytic mechanism. This difference in inhibitory potency could be attributed to variations in the chemical structure of these compounds, influencing their binding affinity and mode of interaction with MAGL. To quantify the inhibitory potency of the most effective compounds, the IC50 values for compounds **3** and **4** were also determined. The significantly lower IC50 value for compound **4** (6.5 µM) compared to compound **3** (21 µM) indicates that compound **4** is 3-fold more potent in inhibiting MAGL (Figure S2). This higher potency could be due to a more favorable binding pose within the enzyme’s active site, stronger binding interactions, or a combination of both.

Having established the inhibitory nature of the proposed compounds, we sought to investigate the reversibility of their interaction with MAGL. Dilution experiments were performed to determine whether the inhibition was reversible or irreversible.

**Figure 4:**
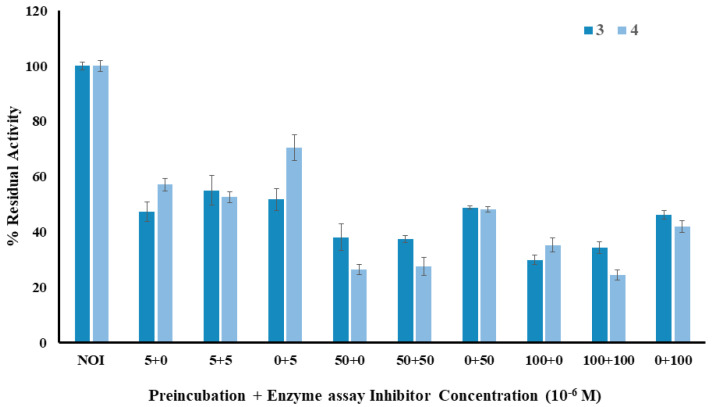
Reversibility of inhibition of compound **3** and **4**. NOI = 100% MAGL activity (experiment performed in absence of inhibitors), for others the first number represents the concentration of first inhibitor addition and the second number the addition of inhibitor after dilution (i.e., 5 + 0 = 5 µM initial concentration, no addition of inhibitor after dilution).

To evaluate the reversibility of inhibition, compounds **3** and **4** were incubated with *h*MAGL at three concentrations—low, medium, and high—to evaluate the reversibility at very low, medium, and high inhibitions of enzyme activity, followed by 15-fold dilution. Additional amounts of inhibitors were added and incubated for another 20 min before initiating the reaction with 7-HRO. The results showed no changes in residual activity after the second addition, indicating irreversible inhibition (Figure 4).

The results of these experiments clearly demonstrate that the inhibitory effects of compounds **3** and **4** are irreversible. The inhibition indeed was mostly determined by the initial concentration of compounds **3** and **4**, rather than by the second addition. This lack of activity recovery strongly suggests that compounds **3** and **4** form a stable, irreversible complex with MAGL. This irreversible binding could involve covalent modification of the enzyme, or a very tight, non-covalent interaction that effectively prevents substrate binding and catalysis. The formation of such a stable complex would explain the observed reduction in MAGL activity and the lack of HDI release, which made it impossible to use the proposed substrates as CL probes.

**Figure 5:**
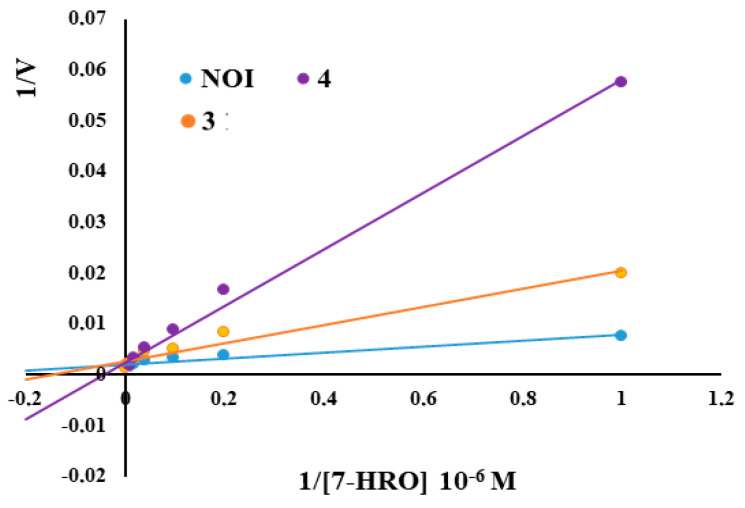
Double reciprocal plots of the inhibition kinetics of *h*MAGL by compound **3**–**4**. 7-HRO concentrations: 1–5–10–25–50–100 µM, NOI (experiment performed in absence of inhibitor at 50·10^−6^ M).

Finally, to better characterize the inhibition mechanism, fluorescence experiments were performed to determine the inhibition type: competitive, non-competitive, uncompetitive, or mixed. The effects of different concentrations of the substrate 7-HRO with *h*MAGL in the presence or absence of compounds **3** and **4** were analyzed. Michaelis–Menten curves gave Vmax and Km values and a Lineweaver–Burk plot was built reporting 1/V vs. 1/[7-HRO] (Figure 5). The obtained data (reported in Supplementary Materials, Table S1), with a not significant variation in Vmax and a 4- or 6-fold increase in Km, suggest a competitive inhibition for both molecules.

Moreover, a preliminary experiment aiming to assess the selectivity of compounds **3**–**4** was performed with the fatty acid amido-hydrolase (FAAH), the principal enzyme involved in endocannabinoid hydrolysis, together with MAGL. A well-known fluorescence method using the fluorogenic substrate 7-amino-4-methyl Coumarin-Arachidonamide (AAMCA) as a probe to evaluate FAAH activity was used, as reported in the literature [28]. The preliminary results (Supplementary Figure S3) show a slight inhibitory activity of compound **4** on FAAH at high concentrations (100 µM).

Further investigation will be needed to elucidate the precise molecular mechanism of this irreversible, competitive inhibition, including identifying the specific residues involved in the interaction and determining the nature of the binding event.

### 2.4. Docking Studies

To investigate the interactions between the lophine esters and human MAGL, molecular docking calculations were performed. Table 1 displays the numerical values for all calculated “scoring” functions associated with different ligand–amino acid interactions. In the evaluation of inhibitory potency, the consideration of various scoring functions is essential due to their associations with distinct interactions. The results indicate that molecule **4** exhibited the highest potential interactions based on the MolDock and ReRank “score” functions, with **2** demonstrating the lowest. Similarly, the Energy “score” function, representing the sum of all energies related to ligand–active site interactions, ranked molecule **4** highest and **2** lowest. Regarding HBond and NoHBond90 “score” functions, which account for hydrogen bonds between ligands and amino acids at the enzyme active site, molecule **3** displayed the highest energies, while 4 showed the lowest. In terms of Van der Waals interactions within the active site (VdW “score” function), molecule **3** exhibited the highest energies, contrasting with **4**, which displayed the lowest.

KDeep is a web-based tool that calculates absolute binding affinity between proteins and ligands [54,55]. In earlier docking experiments, the molecules were ranked using a relative criterion of interaction affinity. The accuracy of this ranking is contingent upon deriving the corresponding free energy values for the interaction of candidate molecules with the receptor. The binding affinity results obtained using the KDeep docking tool are summarized in Table 1. The calculated binding free energies (ΔG) and ligand efficiencies (LE) are expressed in kcal/mol, while the predicted binding affinities (pKd) provide insight into the relative strength of ligand–receptor interactions. Among the tested compounds, **3** demonstrated the highest binding affinity, with a ΔG of −10,368 kcal/mol, and the highest predicted pKd value of 7.6801, indicating the strongest predicted binding to the receptor. A higher pKd value suggests a lower dissociation constant (Kd) and thus a stronger and more stable ligand–receptor complex. Compound **4** showed a comparable binding affinity with a ΔG of −9.9003 kcal/mol and a pKd value of 7.3336, suggesting moderately strong binding. Interestingly, although **2** had a slightly less favorable binding energy (ΔG = −9.599 kcal/mol), its predicted pKd value of 7.1103 indicates that it still forms a reasonably stable complex with the receptor. Importantly, the trend in predicted pKd values closely matches the experimental activity data for these ligands, confirming the reliability of the KDeep-based predictions. The strong correlation between computational and experimental results further supports the accuracy of the model and its potential to predict binding strength and complex stability with high confidence.

**Figure 6:**
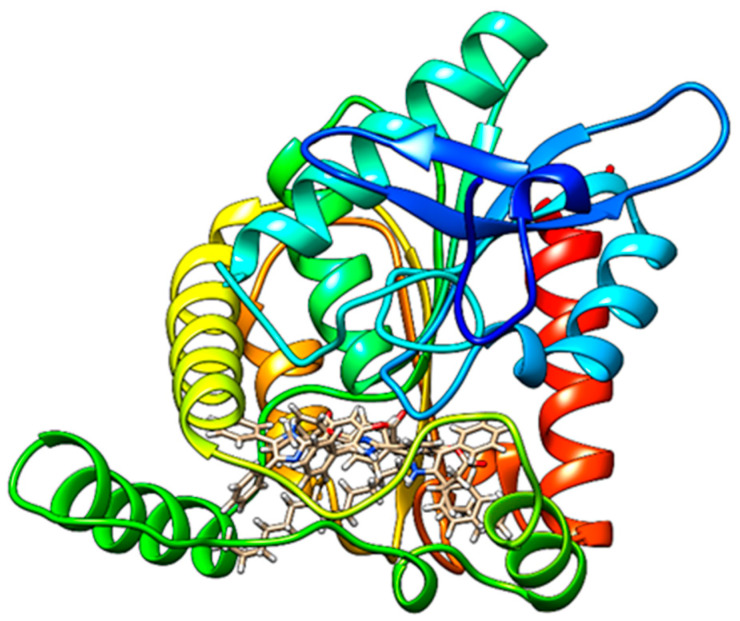
The best calculated poses for all studied molecules within the *h*MAGL active site.

These findings provide insights into the varying interaction profiles of the tested molecules with *h*MAGL, with molecule **4** demonstrating the most favorable overall interactions in most scoring categories. Figure 6 illustrates the best calculated poses for all designed molecules within the active site of *h*MAGL.

Molecule **2** forms a hydrogen bond with Met123, π-π interactions (stacking and T-shaped) with His269 and Tyr194, a π–sigma interaction with Val270, and an unfavorable steric clash with Arg57. Molecule **3** forms hydrogen bonds with Arg57 and Ser122, along with a π–sigma interaction with Ala51. Molecule **4** forms a hydrogen bond with Glu53 and an unfavorable steric clash with Ala51, which had previously formed a π–sigma interaction with **3**. The interactions between the studied molecules (**2**–**4**) and amino acids at the human MAGL active site are comprehensively described, with 2D representations of hydrogen bonds, as well as hydrophobic and hydrophilic interactions inside the binding pocket presented in the Supplementary Materials (Figures S4–S6).

## 3. Materials and Methods

### 3.1. Chemistry

#### 3.1.1. General Information

All reagents and solvents used were of synthetic or analytical grade and were purchased from Merck (Darmstadt, Germany). Deionized water was prepared using a Milli-Q Simplicity 185 filtration system from Millipore (Bedford, MA, USA). Solvents were dried using standard methods and distilled prior to use. The progress of all reactions was monitored by thin-layer chromatography (TLC) on Merck 60 F254 silica gel plates. Flash chromatography was performed with normal-phase silica gel (230–240 mesh, Merck, Darmstadt, Germany).

Nuclear magnetic resonance (NMR) spectra were recorded at 298 K on a Bruker (Manning Park Billerica, MA, USA) AM-500 spectrometer equipped with a 5 mm inverse-geometry broadband probe, operated at 500.13 MHz for ^1^H. Chemical shifts are reported in parts per million and are referenced to the solvent residue proton signal for ^1^H (*δ* = 7.26 ppm for CDCl_3_; *δ* = 3.31 ppm for CD_3_OD; *δ* = 2.50 ppm for DMSO-*d6*). Coupling constants (J) are given in Hertz (Hz), with experimental error in the measured ^1^H–^1^H coupling constants of ± 0.5 Hz. The ^1^H resonances were assigned using correlation 2D ^1^H–^1^H (COSY) experiments. The ^1^H NMR data are presented in the following order: multiplicity (s = singlet, d = doublet, t = triplet, br = broad, m = multiplet), coupling constant(s) in Hz, number of protons, and of proton assignment. These experiments were recorded using a standard pulsed Bruker sequence. The melting point of the molecules was determined using Stuart Scientific SMP3 (Stuart, FL, USA).

*h*MAGL (human recombinant monoacylglycerol lipase) and hFAAH (human recombinant fatty acid amide hydrolase) enzymes were purchased from Cayman Chemical (Ann Arbor, MI, USA). The fluorescent substrate 7-hydroxyresorufyniloctanoate (7-HRO) was synthesized as previously reported [45]. HRP (horseradish peroxidase 150 U/mg) enzyme was purchased from Merck (Darmstadt, Germany).

Fluorometric analyses were carried out in 96-well black plates (Greiner bio-one) using a Jasco FMP 825 fluorometer (W.Yorkshire, UK).

Luminometric analyses were carried out in 96-well, white, clear-bottom plates (Corning) using a Varioskan LUX Multimode Microplate luminometer Reader (Thermo Fisher Scientific, Waltham, MA, USA).

**Figure 7:**
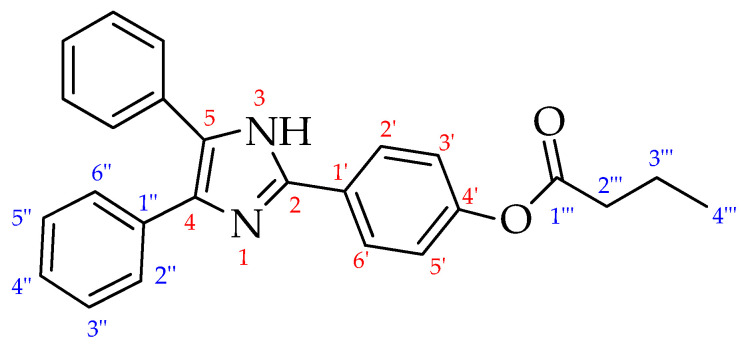
Atom numeration of compound **2** as a general reference for H^1^ NMR assignments.

For the obtained intermediate **1**, and for the final compounds **2**–**4**, the ^1^H NMR assignments refer to the general structure in Figure 7.

#### 3.1.2. 2-(4-Hydroxyphenyl)-4,5-Diphenylimidazole (**1**) [50]

In a round-bottomed flask, diphenylethanedione (benzil) (500 mg, 2.4 mmol), 4-hydroxy benzaldehyde (310 mg, 2.52 mmol), and ammonium acetate (470 mg, 6.1 mmol) were added to acetic acid (6 mL) at 80 °C. The solution was refluxed for 48 h, and the reaction was monitored by TLC (hexane: EtOAc, 97:3, *v*:*v*). Upon completion, the reaction was quenched with ice-cooled water, resulting in a precipitate that was filtered and washed with Et_2_O (10 mL). The crude product was separated from the unreacted starting material by recrystallization from ethanol to give compound **1** (716 mg, 2.3 mmol, 96% yield) as a white solid. mp = 264–265 °C (Lit 264 °C) ^1^H NMR (CD_3_OD): δ 7.83 (2H, d, *J* = 8.8 Hz, 2′ and 6′), 7.48 (4H, d, *J* = 7.0 Hz, 2″ and 6″), 7.34 (4H, t, *J* = 7.0 Hz, 3″ and 5″), 7.30 (2H, t, *J* = 7.0 Hz, 4″), 6.90 (2H, d, *J* = 8.8 Hz, 3′ and 5′).

#### 3.1.3. General Procedure for the Synthesis of Substituted Imidazoles (**2**–**4**)

The appropriate carboxylic acid (1.2 mmol) was dissolved in CH_2_Cl_2_ (6 mL); then, DCC (1.1 mmol) and 4-dimethylaminopyridine (0.64 mmol) were added under an argon atmosphere. After refluxing for 30 min, compound **1** (1.0 mmol) was added. The resulting mixture was stirred for 3 h and monitored by TLC. Upon completion and quenching with water (200 μL), the solution was concentrated under reduced pressure, diluted with CH_2_Cl_2_ (25 mL), and washed with NaHCO_3_ (25 mL) and then brine (25 mL). The organic phase was dried over Na_2_SO_4_ and concentrated under reduced pressure. The residue was purified by column chromatography on silica gel (hexane/EtOAc, 70:30).

#### 3.1.4. Butyric Acid Ester of 2-(4-Hydroxyphenyl)-4,5-Diphenylimidazole (**2**)

Compound 2 was prepared from compound **1** (100 mg, 0.32 mmol) and butyric acid (61 µL, 0.38 mmol) following the general procedure. Purification by column chromatography afforded 88 mg (0.23 mmol, 65% yield) as a white solid. Rf = 0.60 (hexane/EtOAc, 70:30, *v*:*v*). mp = 190 °C. 1H NMR (CD_3_OD) δ 8.02 (2H, d, *J* = 8.2 Hz, 2′ e 6′), 7.49 (4H, d, *J* = 6.8 Hz, 2″ e 6″), 7.34 (4H, t, *J* = 6.8 Hz, 3″ e 5″), 7.30 (2H, t, *J* = 6.8 Hz, 4″), 7.21 (2H, d, *J* = 8.2 Hz, 3′ e 5′), 2.59 (2H, t, *J* = 7.3 Hz, 2″′), 1.82–1.74 (2H, m, 3″′), 1.06 (3H, t, *J* = 7.3 Hz, 4″′).

#### 3.1.5. Octanoic Acid Ester of 2-(4-Hydroxyphenyl)-4,5-Diphenylimidazole (**3**)

Compound 3 was prepared from compound 1 (100 mg, 0.32 mmol) and octanoic acid (61 µL, 0.38 mmol) following the general procedure. Purification by column chromatography afforded 86 mg (0.20 mmol, 63% yield) as a white solid. Rf = 0.725 (hexane/EtOAc, 70:30), mp = 161 °C. ^1^H NMR (CDCl_3_) δ 7.87 (2H, d, *J* = 8.7 Hz, 2′ e 6′), 7.50 (4H, d, *J* = 6.8 Hz, 2″ e 6″), 7.33 (4H, t, *J* = 6.8 Hz, 3″ e 5″), 7.29 (2H, t, *J* = 6.8 Hz, 4″), 7,11 (2H, d, *J* = 8.7 Hz, 3′ e 5′), 2.56 (2H, t, *J* = 7.5 Hz, 2‴), 1.79–1.73 (2H, m, 3‴), 1.36–1.24 (8H, m, 4‴, 5‴, 6‴, 7‴), 0,89 (3H, t, *J* = 7.5 Hz, 8‴).

#### 3.1.6. Palmitic Acid Ester of 2-(4-Hydroxyphenyl)-4,5-Diphenylimidazole (**4**)

Compound 4 was prepared from compound 1 (100 mg, 0.32 mmol) and palmitic acid (98,5 µL, 0.38 mmol) following the general procedure. Purification by column chromatography afforded 120 mg (0.22 mmol, 69% yield) as a white solid. Rf = 0.8 (hexane/EtOAc, 70:30), mp = 146–147 °C (lit: 147–148 °C [56]). 1H NMR (DMSO) δ 8.11 (2H, d, *J* = 8.5 Hz, 2′ e 6′), 7.53 (4H, d, *J* = 6.9 Hz, 2″ e 6″), 7.44 (4H, t, *J* = 6.9 Hz, 3″ e 5″), 7.33 (2H, t, *J* = 6.9 Hz, 4″), 7.21 (2H, d, *J* = 8.5 Hz, 3′ e 5′), 2.96 (2H, t, *J* = 7.3 Hz, 2‴), 1.69–1.63 (2H, m, 3‴), 1.37–1.18 (24H, m, 12 × CH_2_), 0,85 (3H, t, *J* = 7.3 Hz, 8‴).

### 3.2. Biochemistry

#### 3.2.1. Chemiluminescence Experiments

The luminometric assay was performed following established methods from the literature [48,52]. Luminometric tests were conducted in 96-well, white, clear-bottom plates, with each well containing a total volume of 200 µL. The light emission was measured every 2 s for a total duration of 20 min using a Varioskan LUX Multimode Microplate luminometer Reader (Thermo Fisher Scientific, USA). The procedure consisted of two phases: In the first phase, a pro-enhancer is hydrolyzed by MAGL, yielding an active enhancer. This active enhancer then increases the luminescence of the luminol–hydrogen peroxide (H_2_O_2_)–horseradish peroxidase (HRP) system, enabling the CL detection of MAGL activity. Luminol was dissolved in DMSO to give a 0.6 mM stock solution, which was subsequently diluted with DMSO to give a 30 µM solution. *h*MAGL was diluted in the reaction buffer to a working concentration of 2.5 ng/mL. Compounds **1**–**4** were dissolved in DMSO to prepare 5 mM stock solutions, which were then serially diluted to ensure a constant DMSO percentage across all wells. HRP was dissolved in reaction buffer to give a 0.070 mg/mL stock solution and then diluted twofold with the reaction buffer. H_2_O_2_ was used without further dilution. Initial experiments were conducted to optimize the conditions for lophine’s enhancer activity. The second part of the reaction, involving HDI, luminol, HRP, and H_2_O_2_, was first optimized by setting a fixed HDI concentration of 30 µM. The concentrations of HRP been varied from 0.033 unit to 1.7 × 10^−4^ unit, luminol from 3.75 µM to 0.75 µM, and H_2_O_2_ from 10 μL to 2.5 μL. With luminol at 0.9 µM, HRP at 1.7 × 10^−4^ unit, and H_2_O_2_ at 2.5 µL, HDI–luminol ratios of 133:1, 83:1, 55:1, 33:1, 11:1, 5:1, 1:1, 1:4, and 1:8 were then tested.

In the second step, the entire reaction was optimized, focusing on the first part, involving compounds **2**, **3**, **4**, MAGL, and incubation time. The final well concentrations of the molecules tested were 62.5 μM, 30 μM, 15 μM, 10 μM, 5 μM, and 2.5 μM, down to 1 μM, along with varying MAGL amounts from 100 ng to 2.5 ng and incubation times of 0–60 min. A time course analysis was performed with compounds **2**, **3**, and **4** to determine their CL emission profile.

The general procedure used for all pro-enhancers involves an initial incubation of the testing compounds with the enzyme. Compounds **2**–**4** were incubated in microcentrifuge tubes with *h*MAGL in 50 mM tris HCl (pH 7.4) with 1 mM EDTA (reaction buffer). After an incubation period, luminol and H_2_O_2_ are added, in this order, to the same microcentrifuge tubes. The entire contents were then transferred to the well. Before this transfer, HRP was added to each well using a multichannel micropipettor. The plate was placed in the luminometer and read for 20 min, with readings every 2 s. Control experiments were performed to account for the background signal. A blank solution containing luminol, buffer reaction, HRP, and H_2_O_2_ was tested to measure the luminol background signal. Additionally, a blank solution containing the tested molecules (compounds **2**–**4**), luminol, reaction buffer, HRP, and H_2_O_2_ was prepared to assess any possible phenomena of auto-hydrolysis of the molecules.

#### 3.2.2. Fluorescence Experiments

MAGL activity, both with and without compounds **1**–**4**, was evaluated following a published procedure [29], using the fluorescent probe 7-HRO. This substrate is hydrolyzed to octanoic acid and the fluorescent molecule resorufin. Enzyme assays were performed in 96-well black plates, each well containing a total volume of 100 µL. The reaction buffer consisted of 50 mM Tris-HCl (pH 7.4) and 1 mM EDTA. Stock solutions (10 mM) of compounds **1**–**4** and of 7-HRO were prepared by dissolving the solid compounds in DMSO. Eight different working solutions (2 mM, 1 mM, 200 µM, 100 µM, 20 µM, 2 µM, 1 µM, and 200 nM) were then prepared in DMSO from these stock solutions to achieve final well concentrations ranging from 50 nM to 100 µM, while maintaining a constant DMSO percentage across all experiments. A 100 μM working solution of 7-HRO was also prepared by diluting the stock solution in DMSO. In a typical experiment, a 100 µL mixture was prepared in a microcentrifuge tube, consisting of 80 µL of reaction buffer, 12 ng of MAGL dissolved in 10 µL of reaction buffer, and 5 µL of inhibitor solution (or DMSO for condition i). This mixture was incubated for 20 min. Following incubation, 5 µL of the 7-HRO working solution was added to achieve a final concentration of 5 μM in each well. The mixture was then transferred to the well to initiate kinetic experiments. Fluorescence was measured every 2 min for 40 min using a Jasco FMP 825 fluorometer, with excitation and emission wavelengths set at 572 nm and 595 nm, respectively.

Four conditions were tested for each molecule:

i. MAGL with 7-HRO fluorescent probe (inhibitor replaced by 5 µL DMSO, representing 100% MAGL activity).
ii. MAGL in the presence of 7-HRO and the tested molecules.
iii. Blank molecules (10 µL of the enzyme solution replaced by 10 µL of reaction buffer).
iv. 7-HRO fluorescent probe blank solution (10 µL of the enzyme solution replaced by 10 µL of reaction buffer, and inhibitor replaced by 5 µL DMSO).

Three concentrations were selected for the reversibility evaluation. Reversibility working solutions were prepared in DMSO from 10 mM stock solutions to achieve final concentrations of 5 µM (low), 50 µM (medium), and 100 µM (high) in centrifuge microtubes, adding the same amount of DMSO (1 µL) in each. For the experiments, *h*MAGL (12 ng in 10 µL of working buffer) was incubated in centrifuge microtubes with compounds **3** or **4** at the three different concentrations, or with DMSO for “zero” time points, for 20 min. After incubation, the solutions were diluted 15-fold, and new amount of inhibitors were added at varying concentrations (0.33–5 µM, 3.33–50 µM, and 6.66–100 µM for 5–50–100 µM, respectively), and incubated for another 20 min before initiating the reaction with 7-HRO (5 µM final concentration per well). The same procedure was followed for preparing the molecule blank solutions, replacing the enzyme with working buffer and the 7-HRO with DMSO. Fluorescence was then measured every 2 min for 40 min using a Jasco FMP 825 fluorometer, with excitation and emission wavelengths set at 572 nm and 595 nm, respectively.

Experiments to determine the inhibition mechanism were performed using the fluorescent probe 7-HRO in different concentrations according to the method reported in the literature, with slight modifications [57]. The experimental conditions were the same as those used for the activity assessment with MAGL, with the addition of six different concentrations of 7-HRO. In brief, the experimental design involved 96-well black plates, with each well containing a total volume of 100 µL. The reaction buffer consisted of 50 mM Tris-HCl (pH 7.4) and 1 mM EDTA. Stock solutions (10 mM) of compounds **3** and **4** were prepared by dissolving the solid compounds in DMSO. From these stock solutions, 6 different working solutions (2 mM, 1 mM, 500 µM, 400 µM, and 200 µM) were then prepared in DMSO in order to achieve final well concentrations of 25 μM, 50 μM, and 100 μM for compound **3** and 10 μM, 20 μM, and 50 μM for compound **4**. Different working solutions of 7-HRO were also prepared by diluting the stock solution in DMSO. A 100 µL mixture was prepared in a microcentrifuge tube, consisting of 80 µL of reaction buffer, 12 ng of MAGL dissolved in 10 µL of reaction buffer, and 5 µL of inhibitor solution (or DMSO for condition i). Different compound concentrations in the presence or absence of 12 ng of MAGL were pre-incubated for 20 min. Following incubation, 5 µL of the 7-HRO working solution was added to achieve a six different final concentrations of 50 μM, 25 μM, 10 μM, 5 μM, and 1 μM, and the fluorescence was measured after 45 min. The inhibition mechanism of the compounds was analyzed using a Lineweaver–Burk plot generated using the GraphPad Prism 6 program. The inhibition mechanism of the compounds was analyzed using the GraphPad Prism 6 program. Michaelis–Menten curves gave Vmax and Km values, and Lineweaver–Burk plots were generated.

Three concentrations were selected for selectivity evaluation. Enzyme assays were performed as previously described [28] in 96-well black plates, with each well containing a total volume of 200 µL. The reaction buffer consisted of 50 mM Tris-HCl (pH 9) and 1 mM EDTA. Selectivity working solutions were prepared in DMSO from 10 mM stock solutions to achieve final concentrations of 0.1 µM (low), 10 µM (medium), and 100 µM (high) in centrifuge microtubes, adding the same amount of DMSO (1 µL). For the experiments, hFAAH (0.5 U in 50 µL of working buffer) was incubated in centrifuge microtubes with compounds **3** or **4**, or with DMSO as a control blank, for 20 min. After incubation, 2 µL of AAMCA 2 mM working solution was added to achieve a final concentration of 10 μM in each well. The mixture was then transferred to the well to initiate kinetic experiments. Fluorescence was measured every 2 min for 25 min using a Jasco FMP 825 fluorometer, with excitation and emission wavelengths set at 340 nm and 450 nm, respectively.

#### 3.2.3. Data Analyses

Each experiment was performed in triplicate and repeated at least twice. The CL results were analyzed using Microsoft Excel. Means and standard deviations were calculated, and graphs were plotted as relative light unit (RLU) versus time. Fluorescence emission data were processed with Microsoft Excel 2016 and plotted as relative fluorescence units (RFU) versus time. Linear regression was applied for interpolation. The fluorescence emission over time is represented by the course of the different lines. The slopes of the obtained curves were used for comparisons, with the curve corresponding to *h*MAGL without inhibitors set as the 100% value. Means and standard deviations of residual activity percentages were calculated and then represented with a histogram.

### 3.3. In Silico Molecular Docking Simulations

Molegro Virtual Docker (MVD) was utilized for the docking studies in this research [58]. The crystal structure of human MAGL, retrieved from the protein database (PDB: 5zun), served as the target for these studies. The three-dimensional structures of the ligands under investigation were generated using MarvinSketch 6.1.0 with the MMFF94 force field, and their partial charges were calculated employing the Gasteiger–Huckel method. MVD facilitated the analysis of hydrophobic (primarily steric and Van der Waals interactions) and hydrophilic interactions, including the identification of hydrogen bonds between amino acids within the active site and the ligands. This was achieved through docking studies between rigid amino acids of the defined active site and flexible ligands. The interactions were quantified utilizing various scoring functions, such as Hbond, NoHbond, Steric, VdW, Energy, MolDock, and Rerank Score, to provide numerical values indicative of relevant binding energies [59]. The entire molecular docking protocol was validated following an established methodology [60]. The visualization of two-dimensional representations illustrating the interactions between the molecules under investigation and the amino acids in the dopamine transporter active site and receptor surfaces was performed using Discovery Studio Client v20.1.0.19.

The KDeep web-based docking tool utilizes 3D Convolutional Neural Networks (3DCNNs) to enhance the accuracy of binding predictions. It classifies input molecules into eight pharmacophore properties: hydrophobic, aromatic, hydrogen bond donor or acceptor, positive or negative ionizable, metallic, and total excluded volume. The molecules are then processed using a Deep Convolutional Neural Network (DCNN) model trained on the PDBbind 2016 database (available at: https://playmolecule.com/Kdeep/, accessed on 1 June 2024) [5]. The program accepts ligands in ‘sdf’ file format, while the receptor should be provided in ‘pdb’ format. The docking results for ligand orientation within the human MAGL active site obtained with MolDock were used to estimate absolute binding affinity with KDeep.

## 4. Conclusions

This study on the design, synthesis, and evaluation of lophine derivatives successfully presents MAGL activity modulators originating from chemiluminescence studies. While initial investigations focused on identifying chemiluminescence enhancers, our experimental assays surprisingly revealed that none of the synthesized compounds (**2**–**4**) acted as such. Instead, through fluorometric analyses and in silico studies, we uncovered a previously unknown class of MAGL inhibitors. Specifically, compounds **3** and **4** demonstrated potent, concentration-dependent inhibitory effects, and reversibility tests confirmed their stable and irreversible interaction with MAGL. Moreover, kinetic experiments suggest a competitive inhibition type for both molecules, while preliminary experiments on inhibition selectivity toward FAAH showed slight inhibitory activity only for compound 4 at high concentrations. Computational docking studies further elucidated the molecular basis of these findings, showing that compound **4** exhibited the strongest overall affinity for the MAGL active site, while compound **3** exhibited the highest hydrogen bonding and Van der Waals contributions. These studies also highlighted the importance of structural compatibility, as unfavorable steric clashes were observed for certain compounds. In conclusion, this work has not only advanced our understanding of MAGL modulation, but has also serendipitously identified compounds **3** and **4** as promising lead candidates for the development of new lophine-based irreversible MAGL inhibitors. Future research will concentrate on optimizing their structures to maximize binding affinity and selectivity while mitigating steric hindrance within the active site.

## Figures and Tables

**Table 1:** The calculated score values (kcal/mol), ΔG (kcal/mol), and ligand efficiency (LE) (kcal/mol).

Molecule	HBond	NoHBond 90	Steric	VdW	Energy	MolDock Score	Rerank Score	ΔG	LE	pK_d_
**2**	−3.2594	−5.0	−168.812	−21.7531	−148.975	−144.028	−79.9843	−9.5990	−0.2415	7.1103
**3**	−4.786	−7.5	−186.11	−25.6259	−175.106	−161.543	−119.866	−10.3682	−0.3342	7.6801
**4**	−0.1089	−2.5	−174.296	−17.7926	−179.927	−145.218	−106.764	−9.9003	−0.3110	7.336

## Data Availability

The data presented in this study are available at https://doi.org/10.13130/RD_UNIMI/WZLI7H.

## Supplementary Materials

The following supporting information can be downloaded at: https://www.mdpi.com/article/10.3390/molecules30071605/s1, Figure S1. Relative luminescence emitted in the presence of compounds 2-4 using the 33:1 luminol probe, hMAGL, HRP-H2O2; (a) Compound 2 and its blank (b2) without hMAGL; (b) Compound 3 and its blank (b3) without hMAGL; (c) Compound 4 and its blank (b4) without hMAGL. Figure S2. IC50 graph for compounds 3 and 4. Table S1. Kinetic parameters calculated for inhibition mechanism. Table S1. Kinetic parameters calculated for inhibition mechanism. Figure S3. Results of preliminary experiment to assess selectivity of compounds 3 and 4, performed on FAAH enzyme using the fluorogenic probe AAMCA. Figure S4. Two-dimensional representation of the interaction between molecule 2 and amino acids inside monoacylglycerol lipase active site. Figure S5. Two-dimensional representation of the interaction between molecule 3 and amino acids inside monoacylglycerol lipase active site. Figure S6. Two-dimensional representation of the interaction between molecule 4 and amino acids inside monoacylglycerol lipase active site.
