# Supplementary material for: Discovery of MAGL Inhibition by Lophine Derivatives: An Unexpected Finding from Chemiluminescent Assay Development
Source: Molecules. 2025 Apr 3;30(7):1605. doi: 10.3390/molecules30071605 (PMC11990452; doi:10.3390/molecules30071605)
Supplement: Supplementary file 1 [file molecules-30-01605-s001.zip › molecules-3491492-supplementary.pdf]

Supplementary Materials

Discovery of MAGL Inhibition by Lophine Derivatives: An Unexpected Finding from Chemiluminescent Assay Development

Roberta Ottria [1,*], Silvana Casati [1], Ornella Xynomilakis [1], Aleksandar Veselinović [2] and Pierangela Ciuffreda [1]

[1] Dipartimento di Scienze Biomediche e Cliniche, Università degli Studi di Milano, 20157 Milano, Italy; silvana.casati@unimi.it (S.C.); ornella.xynomilakis@unimi.it (O.X.); pierangela.ciuffreda@unimi.it (P.C.)
[2] Department of Chemistry, Faculty of Medicine, University of Niš, Blvd. Dr. Zorana Đinđića 81, 18000 Niš, Serbia; aveselinovic@medfak.ni.ac.rs
* Correspondence: roberta.ottria@unimi.it

Table of contents

Figure S1. Relative luminescence emitted in the presence of compounds **2-4** using the 33:1 luminol probe, hMAGL, HRP- H_2O_2; a) Compound **2** and its blank (b2) without hMAGL; b) Compound **3** and its blank (b3) without hMAGL; c) Compound **4** and its blank (b4) without hMAGL.

Figure S2. IC50 graph for compounds **3** and **4**.

Table S1. Kinetic parameters calculated for inhibition mechanism.

Figure S3. Results of preliminary experiment to assess selectivity of compounds **3** and **4,** performed on FAAH enzyme using the fluorogenic probe AAMCA.

Figure S4. Two-dimensional representation of the interaction between molecule **2** and amino acids inside monoacylglycerol lipase active site.

Figure S5. Two-dimensional representation of the interaction between molecule 3 and amino acids inside monoacylglycerol lipase active site.

Figure S6. Two-dimensional representation of the interaction between molecule **4** and amino acids inside monoacylglycerol lipase active site.

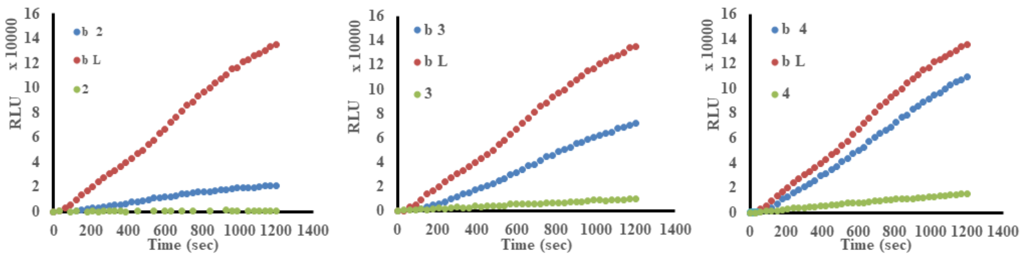

Figure S1. Relative luminescence emitted in the presence of compounds **2-4** using the 33:1 luminol probe, hMAGL, HRP- H_2O_2; a) Compound **2** and its blank (b2) without *h*MAGL; b) Compound 3 and its blank (b3) without *h*MAGL; c) Compound 4 and its blank (b4) without *h*MAGL.

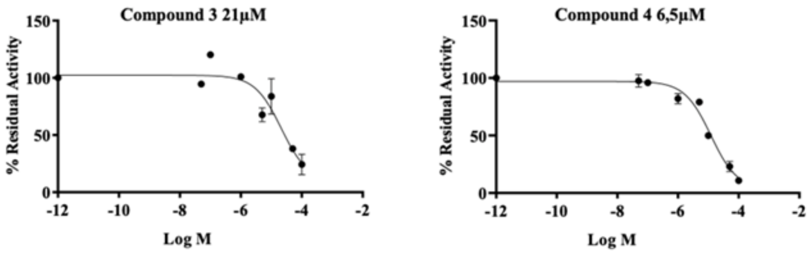

Figure S2. IC50 graph for compounds **3** and **4.**

Table 1. Kinetic parameters calculated for inhibition mechanism.

	NOI	3	4
**Vmax**	777 ± 40	907 ± 94	977 ±67
**Km**	17 ±4	79 ±21	99 ±14
**Kcat**	65	75	81

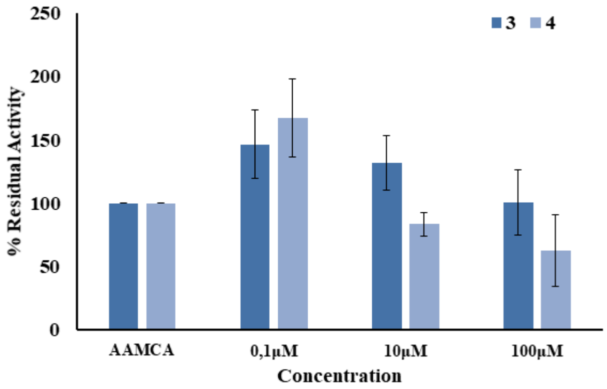

Figure S3. Results of preliminary experiment to assess selectivity of compounds **3** and **4,** performed on FAAH enzyme using the fluorogenic probe AAMCA.

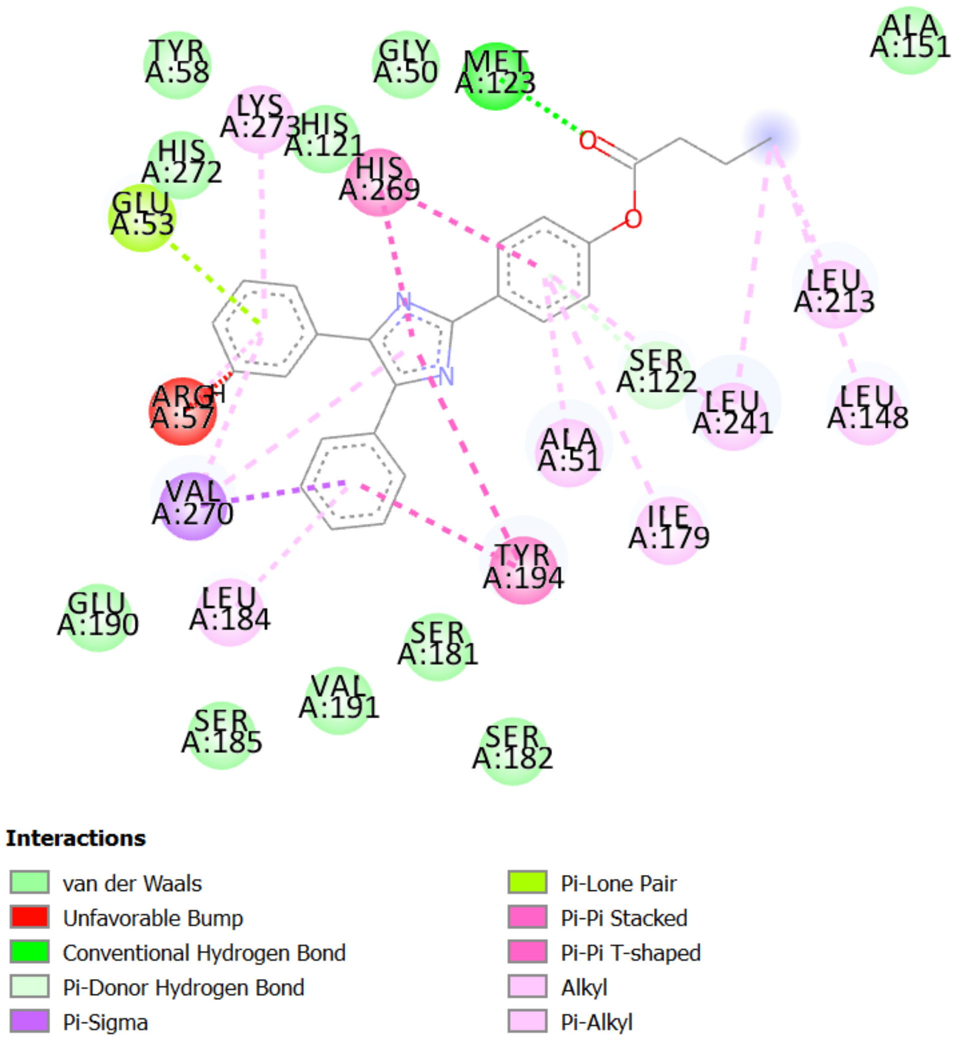

Figure S4. Two-dimensional representation of the interaction between molecule **2** and amino acids inside MAGL active site.

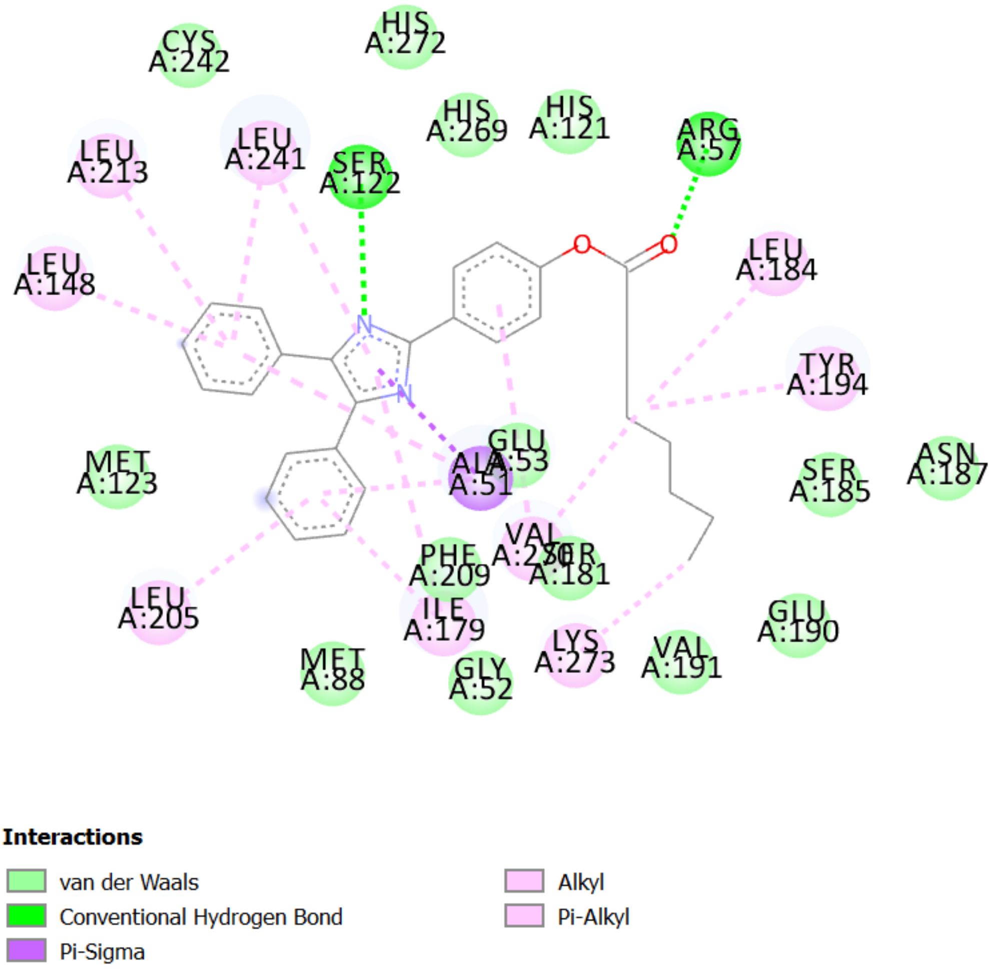

Figure S5. Two-dimensional representation of the interaction between molecule **3** and amino acids inside MAGL active site.

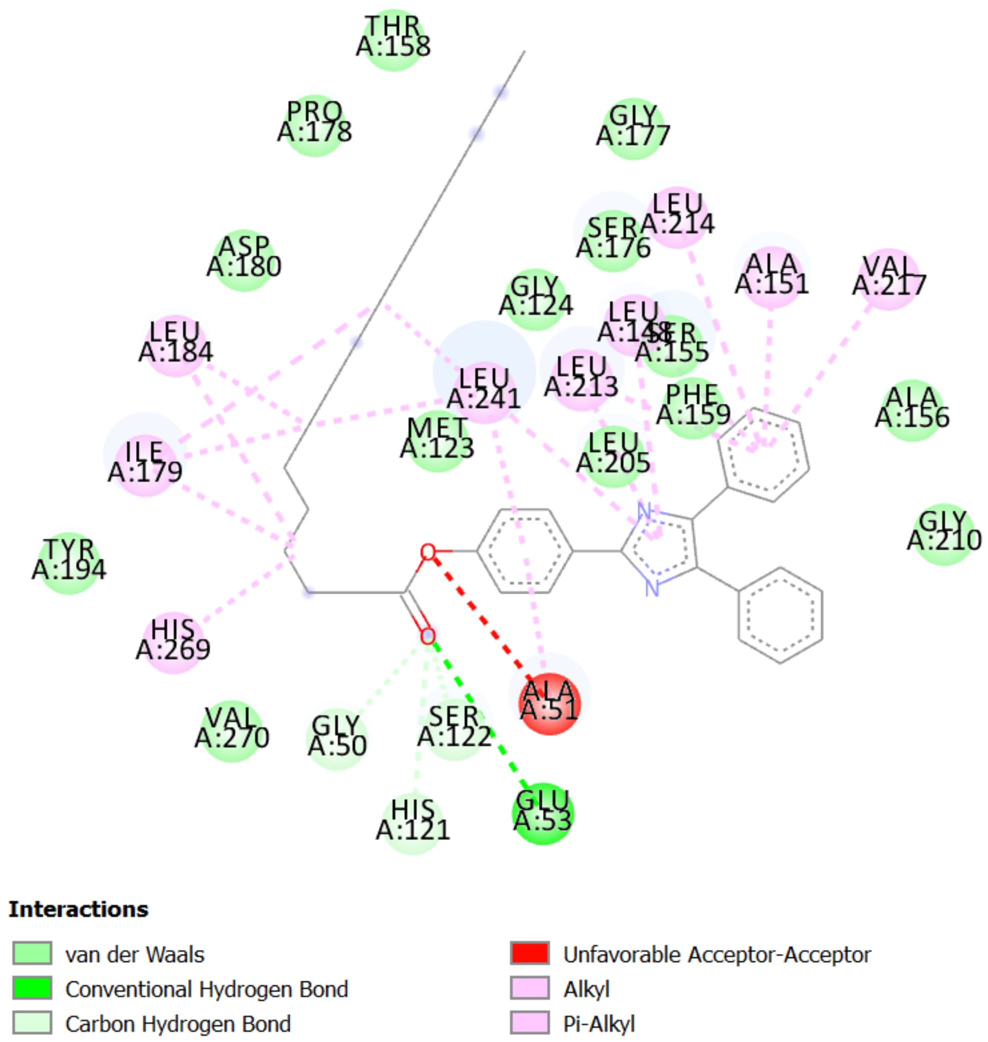

Figure S6. Two-dimensional representation of the interaction between molecule **4** and amino acids inside MAGL active site.
